# Bridging antimicrobial resistance knowledge gaps: The East African perspective on a global problem

**DOI:** 10.1371/journal.pone.0212131

**Published:** 2019-02-11

**Authors:** Frederick K. Wangai, Moses M. Masika, Godfrey N. Lule, Emma M. Karari, Marybeth C. Maritim, Walter G. Jaoko, Beatrice Museve, Antony Kuria

**Affiliations:** 1 Unit of Clinical Infectious Diseases, Department of Clinical Medicine and Therapeutics, School of Medicine, College of Health Sciences-University of Nairobi, Nairobi, Kenya; 2 Department of Microbiology, School of Medicine, College of Health Sciences-University of Nairobi, Nairobi, Kenya; 3 Microbiology Laboratory, Kenyatta National Hospital, Nairobi, Kenya; Eugene Lang College of Liberal Arts at the New School, UNITED STATES

## Abstract

**Background:**

There is worldwide concern of rapidly increasing antimicrobial resistance (AMR). However, there is paucity of resistance surveillance data and updated antibiograms in Africa in general. This study was undertaken in Kenyatta National Hospital (KNH) -the largest public tertiary referral centre in East & Central Africa—to help bridge existing AMR knowledge and practice gaps.

**Methods:**

A retrospective review of VITEK 2 (bioMérieux) records capturing antimicrobial susceptibility data for the year 2015 was done and analysed using WHONET and SPSS.

**Results:**

Analysis of 624 isolates revealed AMR rates higher than most recent local and international reports. 88% of isolates tested were multi-drug resistant (MDR) whereas 26% were extensively-drug resistant (XDR). *E*. *coli* and *K*. *pneumoniae* had poor susceptibility to penicillins (8–48%), cephalosporins (16–43%), monobactams (17–29%), fluoroquinolones (22–44%) and trimethoprim-sulfamethoxazole (7%). *Pseudomonas aeruginosa* and *Acinetobacter baumanii* were resistant to penicillins and cephalosporins, with reduced susceptibility to carbapenems (70% and 27% respectively). *S aureus* had poor susceptibility to penicillins (3%) and trimethoprim-sulfamethoxazole (29%) but showed excellent susceptibility to imipenem (90%), vancomycin (97%) and linezolid (99%).

**Conclusions:**

The overwhelming resistance to commonly used antibiotics heralds a clarion call towards strengthening antimicrobial stewardship programmes and regular AMR regional surveillance.

## Introduction

Antibiotic resistance is estimated to contribute to more than 2 million infections and 23,000 deaths annually in the United States alone, translating to a direct cost of $20 billion and additional productivity losses of $35 billion [1]. Although there is paucity of information on antibiotic resistance in Africa, scanty pockets of data reveal notable rates of antibiotic resistance among extended-spectrum beta-lactamase (ESBL) producers, carbapenem-resistant enterobacteriaceae as well as methicillin-resistant *Staphylococcus aureus*. A systematic look at available continental, regional and finally local data shows a need for concern.

A review of available literature up to the year 2014 documented that the median prevalence of *E*. *coli* and *K*. *pneumonia*e resistance to third-generation cephalosphorins in Subsaharan Africa ranged from 0–47% and 8–77% respectively [2,3]. Regional studies spanning the years 2008 to 2014 revealed that in East Africa, ESBLs were identified in 38–63% of a Kenyan hospital’s samples and 6% of community samples[4]. Meanwhile locally in Kenya, the World Health Organization (WHO) estimated about 60% *E*. *coli* resistance to cephalosporins in incomplete data surveillance in 2012, [2] whereas a publication from a single Kenyan private tertiary hospital reported that between 2007 to 2009, there was 87% *E*. *coli* cephalosporin resistance and between 90 to 92.7% fluoroquinolone resistance among ESBL isolates [5]. For this same hospital, antibiotic susceptibility testing in the year 2014 showed 57% *E*. *coli* resistance to Ciprofloxacin in general [6,7]. There have been similar findings among various Kenyan public health facilities, such as a study done in KNH in 2013 testing pus culture isolates revealing 75.9% *K*. *pneumoniae* resistance to ceftriaxone[8]. A more comprehensive analysis in South Africa revealed 77% third-generation cephalosporin resistance among 923 *K*. *pneumoniae* blood culture isolates in the year 2012 [2].

A few African countries such as Malawi have undertaken long-term surveillance studies demonstrating unmistakable evidence that antibiotic resistance is on the rise in the sub-Saharan Africa region. Between 2003 and 2016, ESBL resistance sky-rocketed up from from 11·8% to 90·5% in *Klebsiella* species, 0·7% to 30·3% in *E*. *coli*, and from 30·4% to 71·9% in other Enterobacteriaceae [9]. Such arduous but worthwhile efforts underscore the need to fill the knowledge gaps on resistance that exist in our own local healthcare facilities, thereby promoting antimicrobial stewardship efforts and curbing of further AMR spread.

## Materials and methods

Kenyatta National Hospital, situated in Nairobi, Kenya, is the largest tertiary and referral centre in East & Central Africa with an estimated 1,800 beds[10]. It registers approximately 89,000 admissions per year[11]. KNH has 50 wards, 22 outpatient clinics and 24 surgical theatres. Of these, eight are adult medical wards with an average of 60 patients per ward at a given time. This study was based in the medical wards and the KNH Microbiology laboratory which processed about 20,693 culture specimens in the year 2015.

This study sought to describe the antimicrobial susceptibility patterns of bacterial isolates from the medical wards in KNH, and thereby build a bridge across existing knowledge and practice gaps relevant to the patient, clinician, hospital and community at large. A one-year review of the antimicrobial susceptibility data of all bacterial isolates cultured from medical ward inpatients was done. These patients are diverse, having interacted with other sectors of the hospital such as surgical, casualty and outpatient clinics. Outpatient clinics encompass various specialties such as medical, diabetic, oncological, general and subspecialized surgical, among others. Furthermore, these patients hail from various community settings from different parts of the country. This study population thereby forms a unique basis of interest as far as antimicrobial susceptibility patterns are concerned, owing to the fact that antibiotic viability is greatly affected by community usage as well as hospital usage. Therefore, the medical wards provided a pool of patients in which we were able to study a mix of community-acquired and healthcare-associated infections from this collective.

### Laboratory testing

Isolates were cultured using standard bacteriological techniques. Identification and antimicrobial susceptibility testing were performed using the VITEK 2 (bioMérieux) system, an automated system used for microbial identification and antibiotic susceptibility testing. It can also perform resistance mechanism detection and aid in epidemiologic trending and reporting. Antibiotics tested included oxacillin (30μg cefoxitin), penicillin G (10 units), clindamycin (2 μg), erythromycin (15 μg), gentamicin (10 μg), tobramycin (10 μg), levofloxacin (5 μg), moxifloxacin (5 μg), linezolid (30 μg), mupirocin, nitrofurantoin (300 μg), rifampicin (5 μg), tetracycline (30 μg), tigecycline (15 μg), trimethoprim/sulfamethoxazole (1.25/23.75 μg), teicoplanin (30 μg) and vancomycin (30 μg). A cut off ≥4 ug/ml for oxacillin testing and positive cefoxitin screening of S. aureus isolates was reported as MRSA as a percentage of out of all S. aureus isolates, as per the CLSI guidelines. The laboratory undertakes periodic external quality assurance through the World Health Organization–National Institute for Communicable Diseases, South Africa (WHO/NICD) and United Kingdom National External Quality Assurance Service (NEQAS).

### Data collection

This study reviewed twelve months’ worth of data that had been previously collected from 1^st^ January to 31^st^ December 2015 in order to report 12-month antimicrobial susceptibility patterns, which is the recommended period of antibiogram reporting as per Clinical Laboratory and Standards Institute (CLSI)[12].

### Data analysis

Data was retrieved from VITEK-2 Antibiotic Susceptibility Testing System (bioMérieux) and imported to WHONET 5.6 software through BACLINK (World Health Organization—WHO). Analysis was done using WHONET, SPSS and Microsoft Excel. All isolates were analysed using the Clinical & Laboratory Standards Institute (CLSI M100-S24) standards[13]. Only the first isolate per organism (species) per patient was included as per CLSI recommendations, and analysis done using CLSI breakpoints. Confidence intervals were calculated using the Agresti-Coull interval, as elaborated in the appendix H of the CLSI M39-A4 document which details analysis and presentation of cumulative antimicrobial susceptibility test data [12].

### Ethics review

Study approval was granted Kenyatta National Hospital/University of Nairobi (KNH/UoN) Ethics and Research Committee. All the data in this study was analysed whilst maximizing patient confidentiality through de-identification and was fully anonymised. Personal identifiers such as patient name were not captured nor disseminated in any format. The Ethics and Research Committee waived the requirement for informed consent for the retrospective data.

## Results

A total of 797 bacterial isolates from the medical wards were reported during the 12 months under study, and 173 of them were excluded due to reasons such as duplicate isolates or missing data, leaving 624 isolates for analysis. Hospital data indicated that the medical wards as a unit contributed the largest proportion of isolates cultured in the laboratory after the paediatrics department, as compared to other units in that year.

### Specimen types

The nine types of specimen collected from the medical wards were urine, pus, blood, pleural fluid, peritoneal fluid, cerebrospinal fluid, sputum, stool and vaginal swabs. Most of the bacteria isolated were cultured from urine (41%), followed by pus (36%) and blood (11%). Fewer isolates were obtained from pleural fluid (6%), peritoneal fluid (3%), cerebrospinal fluid (2%), sputum (1%) and negligible isolates from stool and vaginal swabs.

### Spectrum of gram negative isolates and susceptibility patterns

There were twice as many gram-negative bacteria (419/624, 67%) as there were gram-positive bacteria (205/624, 33%) isolates included in the study. The most frequently isolated gram negative bacteria were *Escherichia coli*, *Klebsiella pneumoniae*, *Proteus mirabilis*, *Pseudomonas aeruginosa* and *Acinetobacter baumanii* in descending order. See Table 1 for isolate listing.

**Table 1:** Gram negative organisms isolated in the study.

SPECIMEN^	*E*. *coli*	*K*. *pneumoniae*	*P*. *mirablis*	*P*. *aeruginosa*	*A*. *baumanii*	*E*. *cloacae*	OTHERS (27 species)**	TOTAL
**Urine**	92	76	5	2	4	6	27	**212**
**Pus**	33	26	26	23	18	5	24	**145**
**Blood**	8	8	1	1	3	2	2	**25**
**Pleural fluid**	6	3	-	1	1	3	7	**21**
**Sputum**	-	6	-	-	-	-	0	**6**
**Peritoneal fluid**	3	-	-	-	-	-	2	**5**
CSF*	-	1	-	-	-	-	1	**2**
**Stool**	-	-	-	-	-	-	2	**2**
**Vaginal swab**	1	-	-	-	-	-	0	**1**
**TOTAL ISOLATES**	**143**	**110**	**32**	**27**	**26**	**16**	**65**	**419**

^ Bacteria isolated from specimens include *Escherichia coli*, *Klebsiella pneumoniae*, *Proteus mirabilis*, *Pseudomonas aeruginosa*, *Acinetobacter baumanii*, *Enterobacter cloacae* among others.

* Cerebrospinal fluid

** Others include insignificant numbers of isolates including *Morganella morganii*, *Serratia fonticola*, *Acinetobacter lwoffii*, *Proteus vulgaris*, *Serratia liquefaciens*, *Serratia odorifera*, *Enterobacter aerogenes*, *Salmonella sp*., *Serratia marcescens*, *Aeromonas hydrophila*, *Citrobacter freundii*, *Raoultella ornitholytica*, *Proteus penneri*, *Pseudomonas putida*, *Alcaligenes faecalis (odorans)*, *Acinetobacter haemolyticus*, *Delftia acidovorans*, *Pantoea agglomerans*, *Ewingella americana*, *Escherichia hermannii*, *Klebsiella oxytoca*, *Klebsiella pneumoniae ss*. *Ozaenae*, *Raoultella planticola*, *Myroides sp*., *Pseudomonas pseudoalcaligenes*, *Shigella flexneri*, and *Yersinia enterocolitica*.

*Escherichia coli*, *Klebsiella pneumoniae* and *Proteus mirabilis* met the threshold for CLSI antibiogram reporting, that is, more than 30 isolates per species [12]. Although *Pseudomonas aeruginosa* and *Acinetobacter baumanii* had less than 30 isolates each, their AST results have been described below due to their high clinical significance (see Table 2). The results indicated that the *E*. *coli* and *K*. *pneumoniae* had poor susceptibility to penicillins (8–48%), cephalosporins (16–43%), monobactams (17–29%), fluoroquinolones (22–44%) and trimethoprim-sulfamethoxazole (7%). *E*. *coli* had moderate susceptibility to nitrofurantoin (56%). Both *E*. *coli* and *K*. *pneumoniae* had high susceptibility to meropenem (76–87%) and excellent susceptibility to amikacin (91–97%). *Proteus mirabilis* showed poor susceptibility to cefuroxime (34%) and trimethoprim-sulfamethoxazole (9%); moderate susceptibility to ampicillin-sulbactam (59%), cefepime (53%), ceftriaxone (50%), gentamicin (53%); and high susceptibility to amoxicillin-clavulanic acid (81%), ceftazidime (75%), aztreonam (81%), and ciprofloxacin (72%). *P*. *mirabilis* showed excellent susceptibility to meropenem (97%) and amikacin (100%).

**Table 2:** Antimicrobial susceptibility of gram negative organisms to various antibiotics.

Gram negative organism	No. of Isolates	PERCENT SUSCEPTIBLE (%S)*														
		PENICILLINS			CEPHALOSPORINS					AMINOGLYCOSIDES		FQ	CPM	OTHERS		
		*AMC*	*AMS*	*TZP*	*CXM*	*CTX*	*CRO*	*CAZ*	*FEP*	*AMK*	*GEN*	*CIP*	*MEM*	*ATM*	*NIT*	*SXT*
*Escherichia coli*	**143**	26	8	48	20	25	25	34	43	97	53	22	87	29	56	7
*Klebsiella pneumoniae*	**110**	27	-	33	16	18	18	17	36	91	31	44	76	17	8	10
*Proteus mirabilis*	**32**	81	59	100	34	44	50	75	53	100	53	72	97	81	-	9
Pseudomonas aeruginosa**	**27**	-	-	56	-	-	-	70	78	89	82	73	70	48	-	-
Acinetobacter baumannii**	**26**	-	23	19	-	8	8	19	19	89	27	23	27	-	-	15
OTHERS (28 species)†	**81**															
**TOTAL**	**419**															

Abbreviations: AMC, Amoxicillin-clavulanate; AMK, Amikacin; AMS, Ampicillin-sulbactam; ATM, Aztreonam; CAZ, Ceftazidime; CIP, Ciprofloxacin; CPM, carbapenems; CRO, Ceftriaxone; CTX, Cefotaxime; CXM, Cefuroxime; FEP, Cefepime; FQ, fluoroquinolones; GEN, Gentamicin; MEM, Meropenem; NIT, Nitrofurantoin; SXT, Trimethoprim-sulfamethoxazole; TZP, Piperacillin-tazobactam

* The percent susceptible for each organism/antimicrobial combination was generated by including the first isolate of that organism encountered on a given patient. Confidence intervals calculated using the Agresti-Coull interval as recommended in the CLSI M39-A4 document.

(-) drug not tested or drug not indicated

** Calculated from fewer than the standard recommendation of 30 isolates

† Others include insignificant numbers of isolates including *Enterobacter cloacae*, *Morganella morganii*, *Serratia fonticola*, *Acinetobacter lwoffii*, *Proteus vulgaris*, *Serratia liquefaciens*, *Serratia odorifera*, *Enterobacter aerogenes*, *Salmonella sp*., *Serratia marcescens*, *Aeromonas hydrophila*, *Citrobacter freundii*, *Raoultella ornitholytica*, *Proteus penneri*, *Pseudomonas putida*, *Alcaligenes faecalis (odorans)*, *Acinetobacter haemolyticus*, *Delftia acidovorans*, *Pantoea agglomerans*, *Ewingella americana*, *Escherichia hermannii*, *Klebsiella oxytoca*, *Klebsiella pneumoniae ss*. *Ozaenae*, *Raoultella planticola*, *Myroides sp*., *Pseudomonas pseudoalcaligenes*, *Shigella flexneri*, and *Yersinia enterocolitica*.

*Pseudomonas aeruginosa* was resistant to amoxicillin-clavulanic acid, ampicillin-sulbactam, cefotaxime, ceftriaxone, cefuroxime and nitrofurantoin. It showed moderate susceptibility to piperacillin-tazobactam (56%) and aztreonam (48%); and higher susceptibility to cefepime (78%), ceftazidime (70%), meropenem (70%), amikacin (89%) and gentamicin (82%). *Acinetobacter baumanii* was resistant to cefuroxime, aztreonam and nitrofurantoin. It had negligible susceptibility to cefotaxime and ceftriaxone. It had poor susceptibility to ampicillin-sulbactam (23%), piperacillin-tazobactam (19%), cefepime (19%), ceftazidime (19%), meropenem (27%), ciprofloxacin (23%), gentamicin (27%) and trimethoprim-sulfamethoxazole (15%). It had high susceptibility to amikacin (89%). See Table 2 for corresponding antibiotic susceptibility patterns.

### Spectrum of gram positive isolates and susceptibility patterns

The most frequently isolated gram positive bacteria were *Staphylococcus aureus*, *Staphylococcus haemolyticus*, *Enterococcus faecalis*, *Staphylococcus epidermidis* and *Enterococcus faecium*. *Staphylococcus aureus* (70 isolates) was the only species that met the threshold for antibiogram reporting (see Table 3). It is likely that a large number of *Staphylococcus haemolyticus*, *Staphyloccus epidermidis* and other *coagulase-negative staphylococcus* species isolated were skin contaminants, and thus their susceptibility rates should be interpreted with caution.

**Table 3:** Gram positive organisms isolated in the study.

SPECIMEN^	*S*. *aureus*	*S*. *haemolyticus*	*E*. *faecalis*	*S*. *epidermidis*	*E*. *faecium*	*Other coagulase negative Staphylococcus*	OTHERS (14 species)**	TOTAL
**Pus**	56	7	10	2	-	-	7	**82**
**Blood**	7	12	1	9	2	2	10	**43**
**Urine**	4	1	9	2	14	1	11	**42**
**Pleural fluid**	2	1	3	3	1	1	5	**16**
**Peritoneal fluid**	-	5	-	4	2	-	3	**14**
CSF*	1	2	2	2	-	-	1	**8**
**Sputum**	-	-	-	-	-	-	-	**-**
**Stool**	-	-	-	-	-	-	-	**-**
**TOTAL ISOLATES**	**70**	**28**	**25**	**22**	**19**	**4**	**37**	**205**

^ Bacteria isolated from specimens include *Staphylococcus aureus*, *Staphylococcus haemolyticus*, *Enterococcus faecalis*, *Staphylococcus epidermidis*, *Enterococcus faecium* among others.

Cerebrospinal fluid

**Others include insignificant numbers of isolates including *Enterococcus gallinarum*, *Staphylococcus sciuri ss*. *lentus*, *Staphylococcus lugdunensis*, *Staphylococcus xylosus*, *Streptococcus pneumoniae*, *Enterococcus casseliflavus*, *Enterococcus durans*, *Staphylococcus saprophyticus ss*. *saprophyticus*, *Staphylococcus capitis ss*. *capitis*, *Staphylococcus intermedius*, *Staphylococcus simulans*, *Staphylococcus cohnii ss*. *cohnii*, *Staphylococcus cohnii ss*. *urealyticum*, and *Staphylococcus warneri*.

The antimicrobial susceptibility testing results indicated that *S*. *aureus* had poor susceptibility to penicillin G (3%), trimethoprim-sulfamethoxazole (29%) and oxacillin (45%) which is a methicillin surrogate. Further cefoxitin screening performed revealed 35 resistant isolates of *Staphylococcus aureus*, thus a calculated prevalence 50% MRSA. Moderate susceptibility was seen to fluoroquinolones (59–61%), macrolides (59–64%), cefuroxime (70%) and gentamicin (78%). Excellent susceptibility was seen to imipenem (90%), vancomycin (97%), linezolid (99%), nitrofurantoin (100%) and quinupristin-dalfopristin (100%).

*Enterococcus faecalis* demonstrated poor susceptibility to tetracycline (16%) and quinolones (44–48%), and moderate susceptibility to imipenem (63%). It had high susceptibility to penicillin G (88%), vancomycin (80%), linezolid (84%), nitrofurantoin (84%) and teicoplanin (84%). Meanwhile, *Enterococcus faecium* was multi-drug resistant to beta-lactam antibiotics, quinolones and aminoglycosides. It demonstrated poor susceptibility to nitrofurantoin (11%) and tetracycline (21%). It showed high susceptibility to quinupristin-dalfopristin (75%), linezolid (90%), vancomycin (95%) and teicoplanin (95%). See Table 4 for the antimicrobial susceptibility patterns of the gram positive isolates.

**Table 4:** Antimicrobial susceptibility of gram positive organisms to various antibiotics.

Gram positive organism	No. of isolates (n)	PERCENT SUSCEPTIBLE (%S)*																			
		PENICILLINS			CEPH	CPM	QUIN		AMINOGLYCOSIDES			MAC		OTHERS							
		*AMS*	*OXA*	*PENG*	*CXM*	*IPM*	*LVX*	*MXF*	*GEN*	*HLS*	*TOB*	*CLI*	*ERY*	*VAN*	*LNZ*	NIT***	*SXT*	*TET*	*MUP*	*QDA*	*TEC*
*Staphylococcus aureus*	**70**	-	45	3	70	90	59	61	78	-	75	64	59	97	99	100	29	51	0	100	97
Staphylococcus haemolyticus**	**28**	67	0	0	33	100	18	18	32	-	36	39	11	89	100	96	7	54	0	100	96
Enterococcus faecalis**	**25**	100	-	88	-	63	44	48	-	100	-	-	-	80	84	84	-	16	-	-	84
Staphylococcus epidermidis**	**22**	-	21	0	67	100	23	23	63	-	84	55	23	100	100	100	36	59	0	100	100
Enterococcus faecium**	**19**	0	-	0	-	0	0	0	-	100	-	-	-	95	90	11	-	21	-	75	95
*Other Staphylococcus*, coagulase negative**	**4**	-	0	0	67	100	25	25	100	-	0	50	0	75	75	100	0	25	0	67	75
OTHERS (15 species)†	**37**																				
**TOTAL**	**205**																				

Abbreviations: AMS, Ampicillin-sulbactam; CEPH, Cephalosporins; CLI, Clindamycin; CPM, Carbapenems; CXM, Cefuroxime; ERY, Erythromycin; GEN, Gentamicin; HLS, Streptomycin-high; IPM, Imipenem; LNZ, Linezolid; LVX, Levofloxacin; MAC, Macrolides; MUP, Mupirocin; MXF, Moxifloxacin; NIT, Nitrofurantoin; OXA, Oxacillin; PENG, Penicillin G; QDA, Quinupristin-dalfopristin; QUIN, Quinolones; SXT, Trimethoprim-sulfamethoxazole; TEC, Teicoplanin; TET, Tetracycline; TOB, Tobramycin; VAN, Vancomycin.

* The percent susceptible for each organism/antimicrobial combination was generated by including the first isolate of that organism encountered on a given patient. Confidence intervals calculated using the Agresti-Coull interval as recommended in the CLSI M39-A4 document.

(-) drug not tested or drug not indicated.

** Calculated from fewer than the standard recommendation of 30 isolates.

*** Suggested interpretation for urine isolates

† Others include insignificant numbers of isolates including *Enterococcus gallinarum*, *Streptococcus pneumoniae*, *Staphylococcus hominis ss*. *hominis*, *Staphylococcus sciuri ss*. *lentus*, *Staphylococcus lugdunensis*, *Staphylococcus xylosus*, *Staphylococcus saprophyticus ss*. *saprophyticus*, *Staphylococcus capitis ss*. *capitis*, *Staphylococcus intermedius*, *Staphylococcus simulans*, *Staphylococcus cohnii ss*. *cohnii*, *Staphylococcus cohnii ss*. *urealyticum*, *Staphylococcus warneri*, *Enterococcus casseliflavus*, and *Enterococcus durans*

### Antibiotic resistance

Nineteen (19) antibiotic class types were tested in total. Resistant bacteria were classified under various concentric categories of non-susceptibility, according to international expert consensus by the European Centre for Disease Prevention and Control (ECDC) and the Centers for Disease Control and Prevention (CDC) [12,14]. Drug resistant (DR) was defined as non-susceptibility to at least one antimicrobial agent. Multi-drug resistant (MDR) was defined as non-susceptibility to at least one agent in three or more antimicrobial categories. Extensively drug-resistant (XDR) was defined as non-susceptibility to at least one agent in all but two or fewer antimicrobial categories (i.e. bacterial isolates remain susceptible to only one or two categories). Possible pandrug-resistant (PDR) was defined as non-susceptibility to all agents in all antimicrobial categories tested. A total of 613 (98%) were drug resistant; 549 (88%) multidrug resistant; 163 (26%) extensively-drug resistant; and 51 (8%) possible pandrug-resistant.

To summarise the clinically important drug-resistant bacteria isolated in our study, the WHO Priority Pathogens List (PPL) released in February 2017 was used[15]. This list contains the 12 most significant antibiotic-resistant bacteria recognised worldwide and the following figures highlight their local prevalence in KNH medical wards as derived from this study. In the critical priority category, we found the following rates of carbapenem resistance: 73% *A*. *baumanii*, 30% *P*. *aeruginosa*, 13% *E*. *coli* [8–19%] and 24% *K*. *pneumoniae* [17–33%]. In the high priority category, we isolated 5% vancomycin-resistant *E*. *faecium*, 3% vancomycin-resistant *S*. *aureus* [0–11%] and 50% Methicillin-resistant *Staphylococcus aureus* (MRSA) [39–61%].

Other notable findings revealed in our study include third-generation cephalosporin (such as ceftriaxone) resistance, for example 75% *E*.*coli* resistance [68–82%] and 82% *K*. *pneumoniae* resistance [73–88%].

## Discussion

The aim of this study was to describe the antimicrobial susceptibility patterns of bacterial isolates from KNH medical ward inpatients. The results presented in this analysis offers a wealth of microbiology and clinical data from the largest tertiary facility in Kenya thus giving the much needed insight into local resistance patterns. By the time of publication, this was the largest clinical study performed in this facility since a laboratory surveillance done from 1991 to 1995[16].

The three bacterial agents of greatest concern in global antibiotic resistance (*E*. *coli*, *K*. *pneumoniae* and *S*. *aureus*) outlined by WHO [2] formed the majority of the bacteria isolated. *E*. *coli* and *K*. *pneumoniae* collectively contributed over 60% of the Gram negative isolates (34% and 26% respectively) whereas *S*. *aureus* formed the bulk of the Gram positive isolates (34%). This spectrum of isolates has also been demonstrated locally [5,7] and internationally [17] in other facilities where these three bacteria were the most common pathogens causing infection. Therefore, these organisms are of important consideration to a clinician when prescribing therapy to treat bacterial infections, especially those caused by gram negative organisms whose outer membrane confers additional resistance to antibiotics as compared to gram positive organisms which lack it[18].

We demonstrated significant rates of antimicrobial resistance to carbapenems, mostly in *A*. *baumanii* (73%) followed by *P*. *aeruginosa* (30%), *K*. *pneumoniae* (24%) and *E*. *coli* (13%). A local private tertiary hospital reported less rates of carbapenem resistance[6] among inpatients in 2014 for *A*. *baumanii* (55%), *P*. *aeruginosa* (15%), *K*. *pneumoniae* (8%) and *E*. *coli* (2%). Differences in resistance rates could be accounted for by differences in hospital infrastructure and patient demographics (higher sociodemographic status with fewer total inpatients) as well as the presence of stronger antibiotic stewardship programmes in the private facility. Meanwhile, the little global data available from WHO is from the region of the Americas and Europe, with some reports of more than 50% resistance to carbapenems in two WHO regions[2].

Cephalosporins, especially the third-generation such as Ceftriaxone and Ceftazidime, were among the most prescribed antibiotics in KNH by most cadres of clinicians and this is reflected as well in other local hospitals [5]. Consequently, there were alarming rates of Ceftriaxone and Ceftazidime resistance reported for *E*. *coli* (75% and 66%) and *K*. *pneumoniae* (82% and 83%) respectively. These rates surpass those seen in other private local facilities for instance one of which registered 49% *E*. *coli* and 61% *K*. *pneumoniae* resistance to Ceftriaxone among inpatients in 2014 [6]. The disparity in antibiotic resistance rates could be possibly attributed to the differences in patient characteristics, disease burden, infrastructure, clinician prescription practices and antibiotic policies between these facilities. Meanwhile, a systematic review of antimicrobial resistance among clinically relevant isolates in sub-Saharan Africa published in 2014 reported median prevalence of third-generation cephalosporin resistance ranging between 0% to 22% in East Africa, between 6% and 15.4% in central South Africa and between 0% and 46.5% in West Africa.[3] This is in contrast to the global estimates of 50% and 30–60% for *E*. *coli* and *K*. *pneumoniae* respectively[2]. Overall, the rates of cephalosporin resistance in this study surpass both regional and global estimates, and this could possibly be fuelled by the indiscriminate prescription of cephalosporins. This underscores the need to explore such and other aggressive drivers of antimicrobial resistance in our setup and their effective mitigation thereof through practices such as informed empirical therapy prescriptions. For instance, nitrofurantoin has been recommended in both local and regional reports to be a favourable option for uncomplicated urinary tract infections caused by E. coli[3,7].

*Staphylococcus aureus* has been known for the last half century to be “notorious” for its ability to rapidly develop antibiotic resistance, since it adapts very well to antibiotic pressure[19]. This was noted with concern in the KNH medical wards, which documented 50% methicillin-resistance among 70 *S*. *aureus* isolates using cefoxitin screening. Reports of MRSA have been on the rise in past studies carried out in various parts of KNH since the 27.7% MRSA rate published in 2003 [20]. The overwhelming high resistance of MRSA in 3 other Kenyan public health facilities was documented in a 2013 publication which reported 84.1% MRSA prevalence through molecular characterisation of the *mecA* gene[21]. The presence of MRSA locally has been augmented by studies involving molecular gene typing of MRSA in both private and public healthcare setups, showing marked genetic diversity and significant presence of epidemic clones locally in Kenya[22]. Although data from Africa is scarce, the WHO 2014 AMR report mentioned national data from 9 African countries ranging between 12–80% [2]. A systematic review of MRSA in Africa published in 2013 documents prevalence as high as 82% in some countries [23].

On the other hand, we speculate that we encountered a possible risk of overestimation of MRSA, through confounding by methicillin-resistant coagulase-negative staphylococcus (CoNS) species misidentified as *S*. *aureus*. CoNS and *S*. *aureus* are frequently isolated together from the same clinical specimen[24]. Misidentification occurs even when using chromogenic agar plates[25] as well molecular Polymerase Chain Reaction (PCR) methods[24]. Since methicillin resistance gene *mecA* is detectable on resistant strains of CoNS as well as *S*. *aureus*, this presents a challenge in true MRSA reporting. False positives have been described [26]. Since molecular detection of *mecA* alone is insufficient for true identification of MRSA, additional *S*. *aureus*-specific gene markers such as *nuc* [24] and *orfX* [26] have to be included during testing. We were unable to carry out molecular testing due to financial constraints.

Overall, our study highlighted the burden of antimicrobial resistance in our setup, especially regarding the critical bacteria in the WHO 2017 priority pathogens list which pose the greatest threat to human health. The rising AMR rates are particularly alarming in the context of low-middle income regions where the new antibiotics developed from the dwindling pipeline in the West are either locally unavailable or unaffordable. Even so, with time these new antibiotics will not be spared from the continuous evolution of resistant bacteria. AMR is best fought by sticking to the first line as much as possible. Ultimately, there emerges a pertinent need for antimicrobial stewardship and continual surveillance locally and at a global scale to protect our antibiotic reserve currently in use.

## Conclusions

This study addressed some key knowledge gaps as pertains to antibiotic sensitivity and resistance patterns in our region, making a significant contribution towards filling the global resistance map. There was overwhelming resistance noted to commonly used antibiotics such as penicillins and cephalosporins. Rising resistance to potent antibiotics such as carbapenems posed a cause of concern. Collaborative efforts involving clinicians with other key stakeholders are needed to strengthen antimicrobial stewardship efforts, and promote regular surveillance and further research towards combating antimicrobial resistance for the present and future generations to come.

## Supporting information

S1 File (XLSX)

## Abbreviations

AMR: Antimicrobial resistance
CDC: Centers for Disease Control and Prevention
CLSI: Clinical Laboratory and Standards Institute
ECDC: European Centre for Disease Prevention and Control
KNH: Kenyatta National Hospital
MDR: multi-drug resistant
MRSA: Methicillin-resistant staphylococcus aureus
PDR: Pandrug-resistant
PPL: Priority Pathogens List
XDR: Extensively-drug resistant
WHO: World Health Organization

## References

[1] Centers for Disease Control and Prevention (CDC). Antibiotic Resistant Threats in the United States. Atlanta; 2013.

[2] World Health Organization. Antimicrobial Resistance. Antimicrobial Resistance: global report on surveillance. Geneva; 2014.

[3] Leopold SJ, van Leth F, Tarekegn H, Schultsz C. Antimicrobial drug resistance among clinically relevant bacterial isolates in sub-Saharan Africa: a systematic review. J Antimicrob Chemother [Internet]. 2014 9 [cited 2016 Mar 25];69(9):2337–53. Available from: http://www.ncbi.nlm.nih.gov/pubmed/24879668 24879668 10.1093/jac/dku176

[4] Storberg V. ESBL-producing Enterobacteriaceae in Africa—a non-systematic literature review of research published 2008–2012. Infect Ecol Epidemiol [Internet]. 2014 1 [cited 2016 Feb 2];4. Available from: http://www.pubmedcentral.nih.gov/articlerender.fcgi?artid=3955770&tool=pmcentrez&rendertype=abstract 10.3402/iee.v4.20342 PMC3955770 24765249

[5] Maina D, Makau P, Nyerere A, Revathi G. Antimicrobial resistance patterns in extended-spectrum β-lactamase producing Escherichia coli and Klebsiella pneumoniae isolates in a private tertiary hospital, Kenya. Microbiol Discov [Internet]. 2013 5 3;1(1). Available from: http://www.hoajonline.com/microbiology/2052-6180/1/5

[6] AgaKhan University Hospital Nairobi, Department of Pathology. Antibiotic Susceptibility Report 2015 (for data through 2014). 2015.

[7] Maina D, Omuse G, Revathi G, Adam RD. Spectrum of Microbial Diseases and Resistance Patterns at a Private Teaching Hospital in Kenya: Implications for Clinical Practice. PLoS One [Internet]. 2016;11(1):e0147659 Available from: http://dx.plos.org/10.1371/journal.pone.0147659 26807811 10.1371/journal.pone.0147659 PMC4726487

[8] Ratemo NK. Antimicrobial susceptibility pattern of acterial isolates from pus samples at Kenyatta National Hospital, Kenya. MSc Dissertation. University of Nairobi; 2014.

[9] Musicha P, Cornick JE, Bar-Zeev N, French N, Masesa C, Denis B, et al Trends in antimicrobial resistance in bloodstream infection isolates at a large urban hospital in Malawi (1998–2016): a surveillance study. Lancet Infect Dis [Internet]. 2017 10 1 [cited 2018 Dec 26];17(10):1042–52. Available from: http://www.ncbi.nlm.nih.gov/pubmed/28818544 28818544 10.1016/S1473-3099(17)30394-8 PMC5610140

[10] Ngumi ZWW. Nosocomial infections at Kenyatta National Hospital Intensive-Care Unit in Nairobi, Kenya. Dermatology [Internet]. 2006 1 [cited 2016 Mar 25];212 Suppl:4–7. Available from: http://www.ncbi.nlm.nih.gov/pubmed/16490968 10.1159/000089192 16490968

[11] Ndegwa LK, Katz M a, McCormick K, Nganga Z, Mungai A, Emukule G, et al Surveillance for respiratory health care-associated infections among inpatients in 3 Kenyan hospitals, 2010–2012. Am J Infect Control [Internet]. 2014;42(9):985–90. Available from: http://www.ncbi.nlm.nih.gov/pubmed/25179331 10.1016/j.ajic.2014.05.022 25179331

[12] CLSI. Analysis and Presentation of Cumulative Antimicrobial Susceptibility Test Data; Approved Guideline—Fourth Edition CLSI document M39-A4. Wayne, PA: Clinical and Laboratory Standards Institute; 2014.

[13] CLSI. Performance Standards for Antimicrobial Susceptibility Testing; Twenty-Fourth Informational Supplement CLSI document M100-S24. Wayne, PA: Clinical and Laboratory Standards Institute; 2014.

[14] Magiorakos A-P, Srinivasan A, Carey RB, Carmeli Y, Falagas ME, Giske CG, et al Multidrug-resistant, extensively drug-resistant and pandrug-resistant bacteria: an international expert proposal for interim standard definitions for acquired resistance. Clin Microbiol Infect [Internet]. 2012 3 [cited 2017 May 26];18(3):268–81. Available from: http://linkinghub.elsevier.com/retrieve/pii/S1198743X14616323 10.1111/j.1469-0691.2011.03570.x 21793988

[15] Kahlmeter G, Singh N. Global Priority List Of Antibiotic-Resistant Bacteria To Guide Research, Discovery, And Development Of New Antibiotics. [cited 2017 Jun 23]; Available from: http://www.who.int/medicines/publications/WHO-PPL-Short_Summary_25Feb-ET_NM_WHO.pdf

[16] Omari MA, Malonza IM, Bwayo JJ, Mutere AN, Murage EM, Mwatha AK, et al Pattern of bacterial infections and antimicrobial susceptibility at the Kenyatta National Hospital, Nairobi, Kenya. East Afr Med J [Internet]. 1997 3 [cited 2016 Mar 25];74(3):134–7. Available from: http://www.ncbi.nlm.nih.gov/pubmed/9185406 9185406

[17] Center for Disease Dynamics Economics & Policy. State of the World’s Antibiotics, 2015. Washington, D. C.; 2015.

[18] Silhavy TJ, Kahne D, Walker S. The bacterial cell envelope. Cold Spring Harb Perspect Biol [Internet]. 2010 5 [cited 2017 Jun 23];2(5):a000414 Available from: http://www.ncbi.nlm.nih.gov/pubmed/20452953 10.1101/cshperspect.a000414 20452953 PMC2857177

[19] Chambers HF, DeLeo FR. Waves of resistance: Staphylococcus aureus in the antibiotic era. Nat Rev Microbiol [Internet]. 2009 9 1 [cited 2017 Jun 24];7(9):629–41. Available from: http://www.nature.com/doifinder/10.1038/nrmicro2200 19680247 10.1038/nrmicro2200 PMC2871281

[20] Kesah C, Ben Redjeb S, Odugbemi TO, Boye CS-B, Dosso M, Ndinya Achola JO, et al Prevalence of methicillin—resistant Staphylococcus aureus in eight African hospitals and Malta. Clin Microbiol Infect [Internet]. 2003 2 [cited 2017 Jun 24];9(2):153–6. Available from: http://linkinghub.elsevier.com/retrieve/pii/S1198743X14629347 12588338 10.1046/j.1469-0691.2003.00531.x

[21] Maina EK, Kiiyukia C, Wamae CN, Waiyaki PG, Kariuki S. Characterization of methicillin-resistant Staphylococcus aureus from skin and soft tissue infections in patients in Nairobi, Kenya. Int J Infect Dis [Internet]. 2013 2 [cited 2015 Dec 23];17(2):e115–9. Available from: http://www.ncbi.nlm.nih.gov/pubmed/23092752 10.1016/j.ijid.2012.09.006 23092752

[22] Omuse G, Van Zyl KN, Hoek K, Abdulgader S, Kariuki S, Whitelaw A, et al Molecular characterization of Staphylococcus aureus isolates from various healthcare institutions in Nairobi, Kenya: a cross sectional study. Ann Clin Microbiol Antimicrob [Internet]. 2016;15(1):51 Available from: 10.1186/s12941-016-0171-z 27647271 PMC5029008

[23] Falagas ME, Karageorgopoulos DE, Leptidis J, Korbila IP. MRSA in Africa: filling the global map of antimicrobial resistance. PLoS One [Internet]. 2013 1 [cited 2015 Nov 2];8(7):e68024 Available from: http://www.pubmedcentral.nih.gov/articlerender.fcgi?artid=3726677&tool=pmcentrez&rendertype=abstract 10.1371/journal.pone.0068024 23922652 PMC3726677

[24] Becker K, Pagnier I, Schuhen B, Wenzelburger F, Friedrich AW, Kipp F, et al Does nasal cocolonization by methicillin-resistant coagulase-negative staphylococci and methicillin-susceptible Staphylococcus aureus strains occur frequently enough to represent a risk of false-positive methicillin-resistant S. aureus determinations by molecular methods? J Clin Microbiol [Internet]. 2006 1 [cited 2017 Jun 29];44(1):229–31. Available from: http://www.ncbi.nlm.nih.gov/pubmed/16390977 10.1128/JCM.44.1.229-231.2006 16390977 PMC1351978

[25] Merz A, Pflüger V, Johler S. Coagulase-negative staphylcococci lead to false-positive results on chromID S. aureus and chromID MRSA agar. Arch Leb. 2014;65:137–40.

[26] French GL. Methods for screening for methicillin-resistant Staphylococcus aureus carriage. Clin Microbiol Infect [Internet]. 2009 12 [cited 2017 Jun 29];15:10–6. Available from: http://linkinghub.elsevier.com/retrieve/pii/S1198743X14606315 10.1111/j.1469-0691.2009.03092.x 19951329

